# Protective effects of recombinant human cytoglobin against chronic alcohol-induced liver disease *in vivo* and *in vitro*

**DOI:** 10.1038/srep41647

**Published:** 2017-01-27

**Authors:** Jian Wen, Yongbin Wu, Wei Wei, Zhen Li, Ping Wang, Shiwei Zhu, Wenqi Dong

**Affiliations:** 1 School of Laboratory Medicine and Biotechnology, Southern Medical University, Guangzhou, Guangdong Province 510515, P.R. China; 2 Department of Hematology, Nanxishan Hospital of Guangxi Zhuang Autonomous Region, Guilin, Guangxi Zhuang Autonomous Region 541002, P.R. China; 3 Department of Clinical Laboratory, Nanxishan Hospital of Guangxi Zhuang Autonomous Region, Guilin, Guangxi Zhuang Autonomous Region 541002, P.R. China

## Abstract

Alcoholic liver disease (ALD) is an important worldwide public health issue with no satisfying treatment available since now. Here we explore the effects of recombinant human cytoglobin (rhCygb) on chronic alcohol-induced liver injury and the underlying mechanisms. *In vivo* studies showed that rhCygb was able to ameliorate alcohol-induced liver injury, significantly reversed increased serum index (ALT, AST, TG, TC and LDL-C) and decreased serum HDL-C. Histopathology observation of the liver of rats treated with rhCygb confirmed the biochemical data. Furthermore, rhCygb significantly inhibited Kupffer cells (KCs) proliferation and TNF-α expression in LPS-induced KCs. rhCygb also inhibited LPS-induced NADPH oxidase activity and ROS, NO and O_2_•^−^ generation. These results collectively indicate that rhCygb exert the protective effect on chronic alcohol-induced liver injury through suppression of KC activation and oxidative stress. In view of its anti-oxidative stress and anti-inflammatory features, rhCygb might be a promising candidate for development as a therapeutic agent against ALD.

Harmful use of alcohol is one of the world’s leading risk factors for morbidity, disability and mortality^1^,^2^. ALD results from the dose- and time-dependent consumption of alcohol^3^,^4^. The progression of ALD is well-characterized and is actually a spectrum of liver diseases, ranging from steatosis, inflammation and necrosis (steatohepatitis), to fibrosis and cirrhosis, and eventually hepatocellular carcinoma (HCC) in some cases^5^. A number of factors are important in the pathogenesis of alcoholic liver injury, including endotoxin, cytokines and other proinflammatory mediators, mitochondrial damage, oxidative stress and immunologic mechanisms^6^,^7^,^8^,^9^. However, there is currently no satisfying treatment available for patients with ALD^10^,^11^,^12^. The development of a safe and effective drug for treatment of ALD is therefore an urgent global health priority.

Although the pathogenesis of ALD has not yet been well elucidated, accumulated evidences have demonstrated that the activation of KCs played a pivotal role in early disease stages^13^,^14^,^15^. KCs, the resident macrophage in the liver, comprise the largest population of resident tissue macrophages in the body. In their primary scavenger role, KCs endocytose foreign particles and bacterial endotoxins, which causes their activation and production of a number of cell signaling and stress pathway modulators, such as reactive oxygen species (ROS) and cytokines, including tumor necrosis factor (TNF)-α and interleukins^16^,^17^. Specifically, TNF-α is a major pro-inflammatory cytokine which is increased in the blood and liver of individuals with ALD. The expression of TNF-α is regulated by the transcription factor nuclear factor kappa B (NF-κB), which is rapidly activated in response to immunologic stimuli such as lipopolysaccharide (LPS) and oxidants^18^. KCs activated by gut-derived LPS release ROS and reactive nitrogen species (RNS), both of which are small, highly reactive molecules that can cause oxidative damage to body cells. Nitric oxide (NO), the main element of RNS and catalyzed by inducible nitric oxide synthase (iNOS), forms much potent oxidants like peroxynitrite. Alternatively, NADPH oxidase (NOX), a major oxidant-generating enzyme expressed in KCs^19^, can contribute significantly to a dramatic increase in release of ROS after ethanol administration^20^,^21^. Once KCs are activated, a large quantity of superoxide anion (O_2_•^−^) via catalyzing the reduction of O_2_ will be greatly generated. O_2_•^−^ can also react with NO to form peroxynitrite (ONOO^−^), another strong oxidizing and nitrating species^22^. The production of large amounts of ROS and RNS gives rise to oxidative stress in liver, in turn resulting in lipid peroxidation of cellular membranes and hepatocyte injury (see Fig. 1). Therefore, agents with hepatic oxidation resistance are thought to be protective against oxidative damage in ALD.

Cytoglobin (Cygb), a fourth member of the globin superfamily in mammals^23^, is a ubiquitously expressed hexa-coordinate globin^24^ and originally identified in hepatic stellate cell of rats^25^. Since its discovery, some unique structural and functional aspects of this protein have been conducted to comprehend. Cygb has been proposed to regulate oxygen content in the cell and deal with hypoxic conditions on the basis of the properties like intrinsic oxygen-binding capacity^26^, NO dioxygenase activity^27^, and ROS scavenging activity^28^. The expression of Cygb increases in response to various stress conditions including hypoxia, oxidative stress and fibrosis^29^. It is known to protect the cells from oxidative stress-mediated injury^30^,^31^ and enhance the survival rate, as demonstrated in oxidant-treated neuronal cell lines, primary hepatic stellate cells, and kidney fibroblasts^32^. The precise mechanisms of the rhCygb actions remain to be pursued. The current study was therefore undertaken to evaluate the protective effects of rhCygb against chronic ALD challenge in rats, and to further explore the underlying mechanisms (Fig. 1) of rhCygb as a promising therapeutic agent.

## Results

### Identification and biological activity assessment of rhCygb

Compared with the negative control, the recombinant protein was highly expressed in IPTG induced E. coli BL21 (DE3). By SDS-PAGE, the detected fusion rhCygb protein band was consistent with the predicted molecular weight of 21.4 kDa, while the same band was not detected in negative (i.e E. coli BL21 (DE3) harboring pET-28a without induction) controls (Fig. 2a). pET-hCyt transformed E. coli were grown and gene expression was induced under optimal conditions. Harvested cells were lysed by sonication and the supernatants were loaded onto affinity chromatography with monoclonal antibody prepared by our laboratory^33^. Bound recombinant protein was eluted and the final preparation gave a single band on the SDS-PAGE gel (Fig. 2b). The purity of rhCygb was 93.3% evaluated by BandScan 5.0 software. To determine the specificity of the antiserum against purified rhCygb, immuno-blot analysis was performed. Western blot analysis showed that the post-induction purified protein reacted with the monoclonal antibody^33^ displaying an expected ~21 kDa band (Fig. 2c). Moreover, as shown in Fig. 2d, the biological activity assessment of rhCygb was measured spectrophotometrically using a T-AOC commercial kits. The antioxidant activity of rhCygb was 95.58 ± 2.67 U/mg (*P* < 0.01) but the controls (SIRT2 and PBS) was 1.23 ± 0.78 U/mg under the same concentrations. hese results indicated that pET-hCyt expression vector was successfully constructed and the recombinant protein could be used for further analysis.

### Histopathological observation of liver tissues from animals with ALD

Our previous study had shown that 30 weeks of ethanol consumption successfully induced a chronic alcoholic liver model^34^. To verify the therapeutic effects of rhCygb on alcoholic liver injury, histopathological examinations of the liver samples were performed by HE staining. Normal diet (ND) group (Fig. 3a,c) showed pathological observationas follows: hepatocytes of similar size, rounded nuclei with clear nucleoli, pink-stained cytoplasm, no infiltration of inflammatory cells in the portal area, orderly arrangement of the hepatic cord in a radial pattern, and normal lobular structure. In marked contrast, rats fed with the ethanol diet (ED) for 40 weeks exhibited distinct changes: a great hepatic fat accumulation in the form of macro- and microvesicles, edema, the under pressure hepatic sinusoid, the disordered hepatic cord, and the unclear lobular structure (Fig. 3b,d). Whereas, these pathologic changes were prevented almost completely by coadministration of rhCygb (3 mg/Kg per day, s.c.) for 10 weeks (Fig. 3e). Similarly, treatment with glutathione (120 mg/Kg per day, s.c.) as a positive control decreased alcohol-induced fatty accumulation but extensive ballooning of hepatocytes were still observed in the liver (Fig. 3f). These results clearly indicate that rhCygb can completely reverse alcoholic steatosis in rats.

### Effects of rhCygb on hepatic biochemical parameters

Levels of liver enzymes (ALT and AST) of ND, ED, EDC and EDG groups are summarized in Table 1. Ethanol-fed rats showed significant higher ALT (44.96 ± 9.44 *vs*. 60.59 ± 10.41) and AST (92.38 ± 14.23 *vs*. 190.46 ± 21.53) activity than that of ND group (*P* < 0.05). Whereas, both the levels of ALT and AST were markedly dropped in EDC group with respect to ED group (*P* < 0.05). There were no significant difference in ALT and AST levels between ND and EDC groups. The level of AST was significantly higher than that in EDC group (*P* < 0.05). Serum lipid profile was also assessed by commercial kits as demonstrated in Table 1, the levels of TG, TC, and LDL-C were markedly elevated in the ED group but were reduced almost equally to the level of ND group after rhCygb treatment (*P* < 0.05), while the serum levels of HDL-C resulted an opposite trend after treatment. Moreover, the rhCygb treatment was significantly (*P* < 0.05) better than the group in which glutathione was used from these biochemical parameters.

### Effects of rhCygb on KC proliferation and TNF-α expression

To assess the protective efficacy of the rhCygb against chronic ALD challenge, rat KCs were cultured andMTT assay (n = 12) was used to detect cell proliferation *in vitro*. Proliferation of cells cultured under 10 μg/mL LPS conditions for 24 h was obvious (*P* < 0.001) (Fig. 4a). The proliferation rates for rhCygb (20 μg/mL) alone and normal control groups presented no significant difference. Compared with the LPS group, the proliferation rates of cells treated with rhCygb from doses of 5 to 20 μg/L were significantly decreased (*P* < 0.05) with the effect of dose-dependent. TNF-α, which plays a pivotal role in the inflammatory cytokine cascade, is also involved in early LPS induced liver injury. Figure 4b shows he effect of rhCygb on LPS-induced TNF-α production in rat KCs. rhCygb (20 μg/mL) alone did not induce much more TNF-α in rat KCs untreated with LPS than the normal control. LPS induced approximately 2.3-fold increase in TNF-α production in rat KCs and this was significantly inhibited by the addition of rhCygb (5–20 μg/mL).

### Effects of rhCygb on LPS-induced oxidative stress

In order to further investigate the possible mechanisms of rhCygb inhibiting oxidative stress, we examined NADPH oxidase activity and ROS or RNS generation. Incubation with rhCygb resulted in a dose-dependent decrease in NADPH oxidase activity (Fig. 5a) and ROS generation (Fig. 5b) compared to the LPS group. In addition, positive fluorescence of ROS was directly observed under the fluorescence microscope (Fig. 5e, upper panels). rhCygb (20 μg/mL) alone and the normal control were negative in the fluorescence assay, while the fluorescence intensity of LPS control was obviously enhanced. Compared with the LPS control, the fluorescence was gradually weakened with the addition of variant doses of rhCygb (5, 10 or 20 μg/mL). Moreover, using DHE and DAF-FM DA, the intracellular superoxide anions (O_2_•^−^) and NO were further quantified. The LPS-induced production of O_2_•^−^ was inhibited by rhCygb in dose-dependent manners (Fig. 5c). The red fluorescence was gradually weakened with the addition of rhCygb (5, 10 or 20 μg/mL) (Fig. 5e, middle panels). Similarly, NO production was increased in the LPS control but decreased significantly after rhCygb (5–20 μg/L) treatment compared with the normal control (*P* < 0.001), as shown in Fig. 5d. The green fluorescence was gradually weakened with the addition of rhCygb (5, 10 or 20 μg/mL) under the microscope (Fig. 5e, lower panels). Together, we hypothesized that the production of NADPH oxidase, ROS, O_2_•^−^ and NO played a key role in the initiation of oxidative stress and experimental ALD. It was also confirmed that rhCygb exerted the protective effect against LPS-induced oxidative stress.

## Discussion

In this study, we evaluated the potential therapeutic efficacy of rhCygb in a rat model of liver disease induced by chronic ethanol exposure. This model makes it possible to achieve pathologic changes in the liver (e.g., steatosis, inflammation, and necrosis) that resemble alterations that occur in the enteral-feeding model without surgery (Fig. 3). In this setting, the levels of ALT, AST, TG, TC, and LDL-C were elevated in the ED group, while the serum levels of HDL-C resulted in an opposite trend (Table 1). These results agree with earlier reports showing that chronic ethanol intake causes dysregulated lipid metabolism^35^,^36^. However, liver injury and steatosis at the histopathological level were completely reversed after rhCygb treatment (Fig. 3). Also, we found that EDC-treated rats showed decreases in serum ALT, AST, TG, TC and LDL-C and increases in serum HDL-C, returning the levels of all six to the ND levels (Table 1). These findings suggest that therapy with rhCygb may be useful in preventing the alcohol-induced liver injury in rats.

Based on the above discussion, the processes *in vivo* by which rhCygb blocked liver injury caused by long-term treatment with ethanol were unclear. As reported previously^37^, KC activation by gut-derived LPS produced pro-inflammatory cytokines and ROS, which were critical components in the initiation and development of ALD. The primary KC was then employed in the present study. Many reports have demonstrated that primary KC has proliferative capacity *in vitro*^38^,^39^,^40^. Zeng *et al*. have performed several methods to evaluate cell proliferation, including the MTT assay, Propidium Iodide FACS analysis, Ki67 staining^41^. We cited the MTT method to evaluate the primary KCs proliferation and found that the cells did have the ability to proliferate, *in vitro* (Fig. 4a). However, how to activate KCs to achieve the same pathological changes in the gastrointestinal tract? In the pre-experiment, isolated KCs were treated with different concentrations of ethanol, but KCs were found to be very susceptible to apoptosis. Indeed, the best cell is ethanol-fed rats KC. Long-term ethanol exposure leads to bacterial overgrowth in the gut, disruption of intestinal barrier function, and increase in permeability to endotoxin and bacteria. It has been reported that LPS as a bacterial endotoxin has been well established in the role of hepatic inflammation and stimulation of innate immunity^42^,^43^. Therefore, *in vitro* treatment of KC directly with LPS can achieve the same effect with KC from ethanol-fed rats. In this work, we directly used LPS (10 μg/mL) to stimulate KC activation and induce oxidative stress, and finally our experimental results also confirmed the feasibility of this measure (Fig. 4). Next, the activated KCs were further treated with rhCygb. The results *in vitro* revealed that rhCygb abrogated the KC proliferation and TNF-α expression to a level comparable to the control (Fig. 4), which confirmed the results obtained *in vivo*.

Oxidative stress can be mediated by an increase in ROS/RNS production, by a decrease in the antioxidant defense, or an increase in the reactivity of ROS/RNS^44^. The intracellular ROS, NO and O_2_•^−^ levels were measured in our cell models, which presented low levels in normal control group (Fig. 5b–d). However, owing to the potential function of these molecules also to damage normal tissue, the balance between pro-oxidants and antioxidants was crucial for the survival and function of aerobic organisms. If the balance is tipped to favor overproduction of these species, oxidative stress can occur. In support of this hypothesis, KCs that were incubated with 10 μg/mL LPS for 24 h exhibited a 3~5 fold increase of ROS, NO and O_2_•^−^ production in LPS alone group (Fig. 5b–d). Alternatively, oxidant-generating enzymes (e.g., NADPH oxidase, iNOS) in KCs might contribute significantly to a dramatic increase in release of ROS/RNS after ethanol administration^19^. As shown in Fig. 5a, the activity of NADPH oxidase was enhanced by LPS administration.

**Figure 1:**
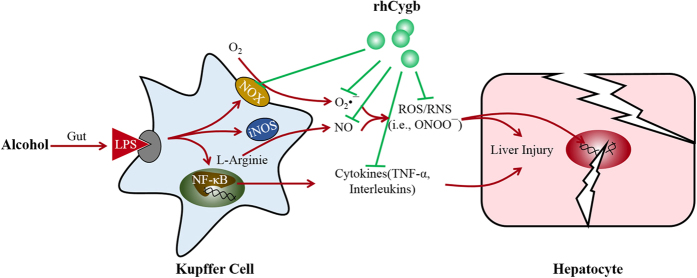
The potential mechanism of rhCygb on alcohol - induced liver injury. Kupffer cells activated by gut-derived LPS release ROS, RNS and inflammatory cytokines (e.g., TNF-α), thus give rise to oxidative stress in liver, resulting in lipid peroxidation of cellular membranes and hepatocyte injury and even DNA damage. Based on this working model, rhCygb was employed to prevent or reverse the liver injury. (Supplementary Figure 1)

**Figure 2:**
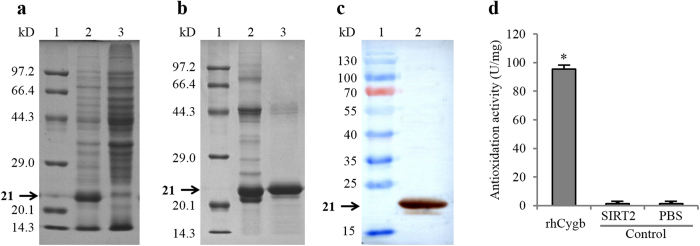
rhCygb identification profile and biological activity assessment. (**a**) SDS-PAGE analysis of rhCygb expression: Lane 1, Protein marker; Lane 2, Induced E. coli cell lysate; Lane 3, Uninduced E. coli cell lysate clone. (**b**) Purification of rhCygb: Lane 1, Protein marker; Lane 2, Purified protein by sephadex G-25; Lane 3, Purified protein by affinity chromatography. (**c**) Western blot analysis of purified rhCygb: Lane 1, Protein marker; Lane 2, Purified rhCygb; Lane 3, Cygb knockout cell lysate. (**d**) Biological activity assessment of rhCygb: The activity of rhCygb was significantly (*P < 0.01) higher than the controls (SIRT2 and PBS) at the same concentrations. (The gel of Fig. 2a,b has been cropped, the full-length gels are presented in Supplementary figure 2a and Fig. 2b. The blot of Fig. 2c has not been cropped, the original blot is presented in Supplementary Figure 2c).

**Figure 3:**
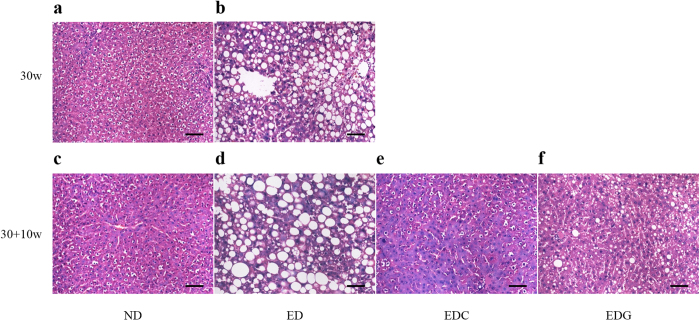
Hepatic histopathological analysis. Representative photomicrographs of HE staining for observing the morphology of livers from different groups (×200). (**a,c**) The normal diet (ND) group, normal histoarchitecture of the hepatic tissues. (**b,d**) The ethanol diet (ED) group, increased hepatocyte steatosis with inflammation and ballooning. (**e**) The ED + rhCygb (3 mg/kg, EDC) group, decreased fatty degeneration and regeneration in hepatocytes. (**f**) The ED + glutathione (120 mg/kg, EDG) group, decreased fatty accumulation but extensive ballooning in hepatocytes. The scale bar represents 25 μm.

**Figure 4:**
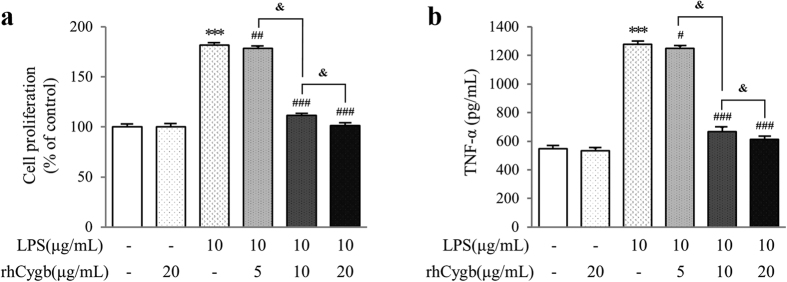
Inhibition of LPS-induced cell proliferation **(a)** and TNF-α production **(b)** by rhCygb. Data are expressed as mean ± S.D. (n = 12). ***P < 0.001 *vs*. normal control; and ^#^*P* < 0.05, ^##^*P* < 0.01, ^###^*P* < 0.001 *vs*. LPS control; ^&^*P* < 0.05 *vs*. 5, 10 μg/mL rhCygb.

**Figure 5:**
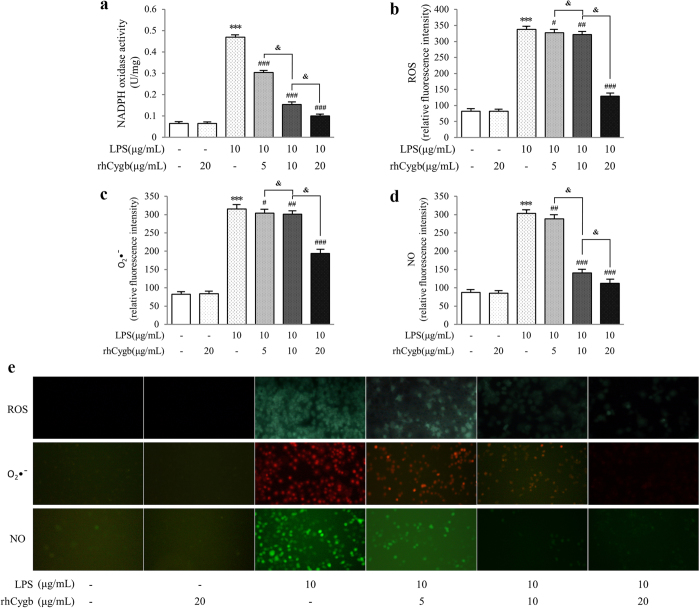
Effects of rhCygb on NADPH oxidase activation, ROS, O_2_•^−^ and NO generation in rat Kupffer cells. (**a**) NADPH oxidase activity. Cultured KCs were treated with LPS (10 μg/mL) in the presence or absence of variant doses of rhCygb (0, 5, 10 or 20 μg/mL) for 24 h. NADPH oxidase activity was then measured. Data are expressed as mean ± S.D. (n = 6). ***P < 0.001 *vs*. normal control; ^###^*P* < 0.001 *vs*. LPS control; ^&^*P* < 0.05 *vs*. 5, 10 μg/mL rhCygb. (**b–e**) Intracellular productions of ROS, O_2_•^−^ and NO. Intracellular ROS levels were assessed using a fluorescent probe, DCFH-DA. Fluorescence intensity was detected by multimode reader (**b**) and observed directly under the fluorescence microscope (**e** upper panels). The light green fluorescence represented intracellular ROS. Intracellular O_2_•^−^ was detected by a fluorescent probe, DHE. Fluorescence intensity was detected by multimode reader (**c**) and observed directly under the fluorescence microscope (**e**, middle panels). The red fluorescence represented intracellular O_2_•^−^. Intracellular NO was detected by a fluorescent probe, DAF-FM DA. Fluorescence intensity was detected by multimode reader (**d**) and observed directly under the fluorescence microscope (**e**, lower panels). The green fluorescence represented intracellular NO. Data are expressed as mean ± S.D. (n = 12). ***P < 0.001 *vs*. normal control; ^#^*P* < 0.05, ^##^*P* < 0.01, ^###^*P* < 0.001 *vs*. LPS control; ^&^*P* < 0.05 *vs*. 5, 10 μg/mL rhCygb.

Cygb is thought to protect the cells from ROS^45^, free radical-mediated DNA damage^35^, and disruption of cellular functions^31^. However, its functions have not been fully understood, our laboratory has previously reported its effect on hepatic fibrogenesis^46^. Therefore, we tried to determine whether it has a protective effect against ALD according to the anti-fibrotic mechanism. As discussed above, we established an animal model for *in vivo* studies to observe the potential therapeutic efficacy of Cygb, but did not further explore its *in vivo* mechanism. After confirming its efficacy, we next explored its *in vitro* mechanism, mainly focused on LPS-induced oxidative stress pathway (Fig. 1). In our experiment, supplementation with variant doses of rhCygb (5, 10 or 20 μg/mL) could inhibit cell proliferation and reduce the activity of NADPH oxidase as well as decrease the levels of TNF-α, ROS, NO and O_2_•^−^ in a dose-dependent manner (Figs 4 and 5). Fluorescence observation also supported these findings. The results of this study indicate that rhCygb could effectively exert protective effects against LPS-induced oxidative stress and immune injury in rat KCs. These effects may rely on peroxidase activities, nitric oxide dioxygenase of rhCygb, which regulate oxygen and nitric oxide metabolism.

A number of novel targeted therapies for ALD were currently being evaluated in clinical trials. Due to limited self-protection ability, body tissues could not express sufficient Cygb to respond to various stress conditions including hypoxia, oxidative stress and fibrosis. Thus, the provision of an appropriate amount of exogenous Cygb could allow the body to be protected from these conditions. Although its side effects currently precluded the use of rhCygb in clinical settings and require rigors of the drug distribution program and prescription decision, efforts to synthesize rhCygb analogues lacking teratogenic effects but having more potent efficacy are underway. Taken together, rhCygb might be a promising candidate for development as a therapeutic agent against ALD and other diseases caused by oxidative damage as well as excessive inflammatory response.

## Methods

### Reagents

Fetal bovine serum, RPMI 1640 medium, trypsin-EDTA (0.25%) were obtained from Hyclone (Logan, UT, USA). Hematoxylin-Eosin (HE), Lipopolysaccharides (LPS), Methyl thiazolyl tetrazolium (MTT), dimethyl sulfoxide (DMSO) and isopropyl-β-D-thiogalactoside (IPTG) were obtained from Sigma-Aldrich (MO, USA). Bicinchoninic acid assay (BCA protein assay) kit was purchased from Beyotime (Jiangsu, China). Assay kit for the total antioxidant capacity assay (T-AOC assay) was purchased from Nanjing Jiancheng Bioengineering Institute (Nanjing, China). Glutathione was purchased from YaoPharma, Co., Ltd., (Chongqing, China). All of the other chemicals utilized in this study were of analytical grade, and the chemicals used for electrophoresis were obtained from Bio-Rad (CA, USA).

### Preparation of rhCygb

rhCygb was expressed in E. coli and purified according to previously established methods, and then desalted with sephadex G-25 resin^46^. The purified protein was analyzed with 12% SDS-PAGE and identified by Western blot experiments. The yields of rhCygb were measured by the software BandScan 5.0 based on SDS-PAGE results. The total antioxidation capacity (T-AOC) of rhCygb was measured spectrophotometrically using a T-AOC commercial kits (Nanjing Jiancheng Bioengineering Institute, Nanjing, China) along with human sirtuin 2 (SIRT2, negative control) and protein dilutions of PBS (blank control). The acute toxicity of rhCygb was determined *in vivo* using the procedure approved by Li *et al*.^46^.

### Animals and treatments

**Figure 6:**
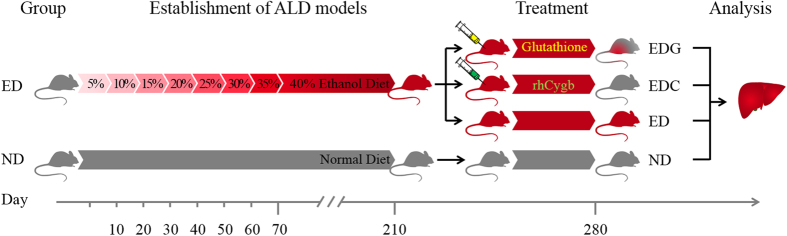
Experimental design of ALD model and animal treatment. Rats in ED group were administered a liquid diet containing ethanol to establish chronic ALD models. The 5% (v/v) to 35% (v/v) ethanol were supplied for 10 days and the 40% (v/v) concentration for the next 20 weeks. After model was established, they were randomly divided into ED, EDG, and EDC groups for 10 weeks, while the ND group animals received an isocaloric liquid diet containing sugar instead of ethanol throughout the test period. Sera and liver tissues were collected for further analysis.

Animal experimentation was performed according to the guidelines approved by the Chinese Association of Laboratory Animal Care. Male Wistar rats with weight of 125–155 g were obtained from the Experimental Animal Center of Southern Medical University (Guangzhou, China). All rats were randomly group-housed in cages in a temperature-controlled environment (22 ± 2 °C) and humidity (60–70%) with a 12 h light/dark cycle and with free access to chow and water during the acclimation period. For experiment, rats were randomly assigned into ND group and ED group. To induce alcoholic liver disease, the ED-fed rats (n = 80) were administered a liquid diet containing ethanol (liquor), which provided 10% of energy^34^. Liquor (Beijing Red Star Co., Ltd., China) was introduced into the diet by gradually mixing it with distilled water from 5% (v/v) to 35% (v/v) over a 1-week period for adaptation, and then at a concentration of 40% (v/v) for the next 20 weeks. The ND rats (n = 20) received an isocaloric liquid diet containing sugar instead of ethanol. After model was established, the ED-fed rats were randomly divided into ED-only, positive (glutathione, 120 mg/Kg, EDG), and therapy (rhCygb, 3 mg/Kg, EDC) groups (n = 20 per group). The rats were then administered daily subcutaneous injection of the respective treatments for 10 weeks. Body weight was measured once a week during the feeding period. Sera and liver tissues were collected for further analysis after the last injection. The schedule of animal experiments is shown in Fig. 6.

### Pathologic evaluation

All rats were anesthetized and sacrificed. The liver tissues were taken and fixed in 10% neutral formalin, embedded, sectioned at 4 μm, and stained with HE for assessment of steatosis, inflammation, and necrosis. Histopathologic examinations of the liver sections were conducted using Olympus BX41 image system (Olympus Company, Tokyo, Japan).

### Serum biochemical estimation

Blood was isolated from the aorta, centrifuged (300 × g, 4 °C, 7 min), and the serum was stored at −80 °C. 40 μL of individual serum samples was subjected to liver biochemistry evaluation. Aspartate aminotransferase (AST) and alanine aminotransferase (ALT) levels were determined by a Biochemical Analyzer (Konsesyum, Alternative Biomedical Services, Dallas, TX, USA). The levels of triglyceride (TG), total cholesterol (TC), high-density lipoprotein-cholesterol (HDL-C), and low-density lipoprotein-cholesterol (LDL-C) were measured using a biochemical analyzer (Nanjing Jiancheng Bioengineering Institute, Nanjing, China).

### Kupffer cell isolation

KCs were isolated from rat livers by collagenase perfusion as previously described^41^ with little modification. To remove the hepatocytes, the liver cell suspension was centrifuged at 50 × g for 3 min at 4 °C. After that, the KCs remained in the supernatant which was collected and moved into another 50 ml centrifuge tube for centrifuging and washing several times. Then, the cell suspension was gently layered onto the 25% and 50% Percoll and centrifuged at 800 × g for 15 min at 4 °C. Lastly, the cell suspension was divided into four layers and the KCs stayed in the third layer. We collected the third layer to centrifuge to get the target cells. And then the cells were cultured for 2 h in 6 well plastic plates (2 × 10^6^ cells/ml) in 5% CO_2_ air atmosphere at 37 °C. The KCs were separated from other nonparenchymal cells by their ability to adhere to uncoated plastic. The purity of the KC cultures was more than 95% as judged by the phagocytosis of India ink.

### Cell culture and treatments

Rat KCs were plated in RPMI 1640 medium supplemented with 10% fetal bovine serum at 37 °C and 5% CO_2_. After KCs attachment, the cells were gently washed with fresh medium to remove unattached cells every 2–3 days. The cell suspension was prepared by digestion with 0.25% trypsin, and then KCs were seeded at 3 × 10^5^ per milliliter in 96-well plates or 6-well plates. KCs were then treated with LPS (10 μg/mL)^47^,^48^ in the presence or absence of variant doses of rhCygb (0, 5, 10 or 20 μg/mL)^49^ for 24 h. Four repeated wells were set for each group in every experiment, which was repeated three times. After the treatments, the supernatants or cells were harvested for further analysis.

### Cell proliferation assay

Cell proliferation was determined by the 3-(4, 5-dimethylthiazol-2-yl)-2, 5-diphenyltetrazolium bromide (MTT) reduction assay method^41^. In short, cells were cultured in 96-well plates and treated for 24 h as described above. Then, 1 mg/mL MTT solution was added to each well of the plate and incubated at 37 °C under 5% CO_2_ for 4 h. The formazan product was dissolved in dimethyl sulfoxide and the optical density was measured at 570 nm using a multimode reader (Tecan, Austria).

### TNF-α measurement

TNF-α levels were detected from the cell culture media by enzyme-linked immunosorbent assay using a commercially available kit (Dakewe Biotech Co. Ltd., Shenzhen, China) according to the manufacturer’s instructions.

### Measurement of NADPH oxidase activity

NADPH oxidase activity was measured by a quantitative colorimetric assay kit (Genmed Scientific Inc., Shanghai, China). Briefly, KCs were seeded in 6-well plates and treated for 24 h as described above. Thereafter, the protein was extracted from cells and NADPH oxidase activity was evaluated by the kit according to the manufacturer’s protocol.

### Determination of ROS, O_2_•^−^ and NO

The dye 2′, 7′-dichlorofluorescein diacetate (DCF-DA) (Beyotime Institute of Biotechnology, Jiangsu, China) was used to measure changes in ROS levels. Briefly, KCs were incubated with 10 μM DCF-DA for 40 min at 37 °C in 96-well plates, and washed three times with phosphate-buffered saline. Subsequently, ROS levels were determined by analyzing the fluorescence intensity using a multimode reader (Tecan, Austria) and a Nikon fluorescence microscope (excitation/emission, 488/525 nm). Furthermore, intracellular O_2_•^−^ and NO were measured using Dihydroethidium (DHE) and fluorescent probe 4-Amino, 5-aminomethyl-2′,7′-difluorescein, diacetate (DAF-FM DA) (Beyotime Institute of Biotechnology, Jiangsu, China), respectively. DHE (10 μM) or DAF-FM DA (10 μM) was added to cell suspensions which were then incubated at 37 °C for 40 min in the dark. Cells were then washed thrice, resuspended in PBS, and maintained on ice for immediate detection with aforementioned instruments. The excitation/emission wavelength of DHE and DAF were 300/610 nm and 495/515 nm, respectively. The average fluorescent density of intracellular areas was measured to index the O_2_•^−^ and NO levels.

### Statistical analysis

All data were expressed as the mean ± standard deviation from at least 5 independent experiments. The biological activity was analyzed using Student’s t-test. The biochemical experimental data were analyzed by Bonferroni tests. Other analyses were performed by one-way analysis of variance (ANOVA) followed by a Dunnett’s post-hoc analysis. Differences were considered significant if the P-value was less than 0.05. Statistical analyses were performed using SPSS version 13.0 software (SPSS Inc., Chicago, USA).

## Additional Information

**How to cite this article**: Wen, J. *et al*. Protective effects of recombinant human cytoglobin against chronic alcohol-induced liver disease *in vivo* and *in vitro. Sci. Rep.* **7**, 41647; doi: 10.1038/srep41647 (2017).

**Publisher's note:** Springer Nature remains neutral with regard to jurisdictional claims in published maps and institutional affiliations.

## Supplementary Material

Supplementary Information

## Figures and Tables

**Table 1:** Effects of rhCygb on ALT, AST, TG, TC, HDL-C and LDL-C of alcohol-induced liver injury rats.

Group	ALT (IU/L)	AST (IU/L)	TG (mg/dL)	TC (mg/dL)	HDL-C (mg/dL)	LDL-C (mg/dL)
ND	44.96 ± 9.44	92.38 ± 14.23	131.46 ± 15.17	62.08 ± 9.68	52.61 ± 7.87	9.07 ± 2.30
ED	60.59 ± 10.41^a^	190.46 ± 21.53^a^	262.42 ± 12.04^a^	98.36 ± 8.42^a^	27.82 ± 4.41^a^	24.10 ± 3.45^a^
EDC	46.47 ± 7.69^b^	106.01 ± 18.12^b^,^c^	148.21 ± 10.20^b^,^c^	68.30 ± 7.52^b^,^c^	48.11 ± 6.77^b^,^c^	12.18 ± 2.37^b^,^c^
EDG	48.73 ± 6.35^b^	164.07 ± 12.79^a^,^b^	201.24 ± 15.91^a^,^b^	85.60 ± 6.02^a^,^b^	34.91 ± 5.17^a^	20.40 ± 2.05^a^,^b^

Each value represents the Mean ± SD (n = 10).

^a^*P* < 0.05 *vs*. ND group.

^b^*P* < 0.05 *vs*. ED group.

^c^*P* < 0.05 *vs*. EDG group.
