# Supplementary material for: Protective effects of recombinant human cytoglobin against chronic alcohol-induced liver disease in vivo and in vitro
Source: Sci Rep. 2017 Jan 27;7:41647. doi: 10.1038/srep41647 (PMC5269723; doi:10.1038/srep41647)
Supplement: Supplementary Information [file srep41647-s1.pdf]

Supplementary Information

Droplet-based microtumor model to assess cell-ECM interactions and drug resistance of gastric cancer cells

Minjeong Jang[1], Ilkyoo Koh[1], Seok Jae Lee[3], Jae-Ho Cheong[2*], Pilnam Kim[1*]

Supplementary Movie 1. Generation of 300 μm collagen bead

Supplementary Movie 2. Generation of 500 μm collagen bead

Supplementary Movie 3. Generation of 700 μm collagen bead

Movies showing microdroplet generation in microfluidic channel. In flow-focusing droplet generation region, droplets break off from the aqueous phase fluid when shear stress of oil phase fluid overcomes interfacial tension which keeps droplets attached to the aqueous stream. Droplet size and generation rates depend on the combinations flow rate of aqueous and oil phase.

Supplementary Movie 4. AGS cell movement in collagen bead for 3 days.

Supplementary Movie 5. Hs746T cell movement in collagen bead for 3 days.

Additional method

Scanning electron microscopy (SEM) imaging

Collagen beads (4 mg/ml) were washed 3 times in distilled water (DW) for removal of excess salt. And collagen beads were freezed-dried at -80 ºC overnight by using freeze dryer (Ilshin). The dried samples were sputter-coated with platinum. Collagen fibers were viewed by Field Emission scanning electron microscopy (FE-SEM, Sirion, FEI, Netherland) at accelerating voltage of 10.0 kV. SEM images are given in Supplementary Figure S1.

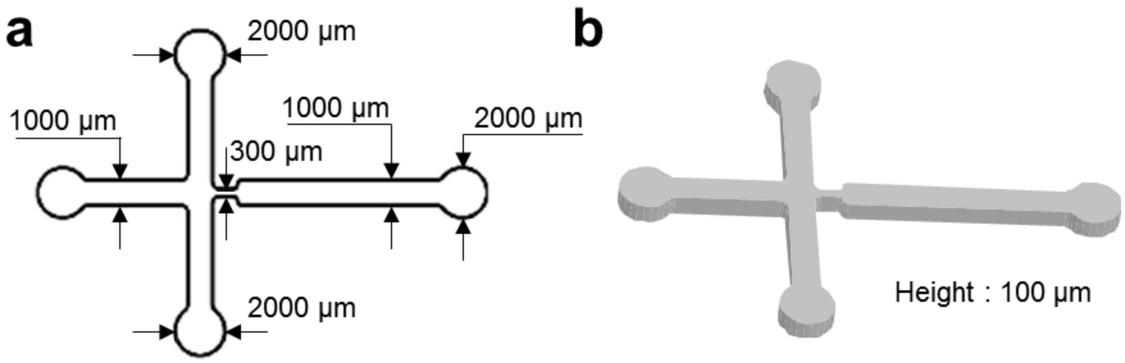

Supplementary Figure S1. Dimensions of microfluidic channel. (a) Schematic diagram of 2D plane channel. Inlet and outlet holes: 2,000 μm. Orifice (cross junction): 300 μm. Three inlet channels and one outlet channel: 1,000 μm. (b) 3D image of microfluidic channel. Channel height: 100 μm.

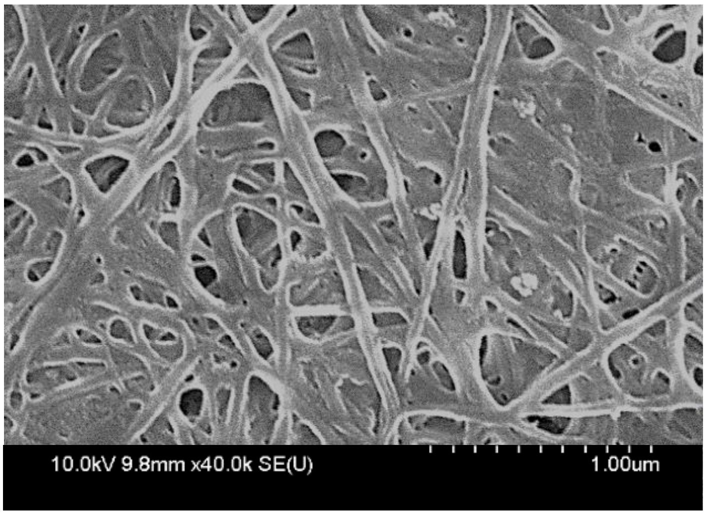

Supplementary Figure S2. Scanning electron microscope (SEM) image of collagen type 1. SEM image of collagen type 1 sample (after gelation with pH 7) at × 40,000 magnification. Collagen fibrils are observed.

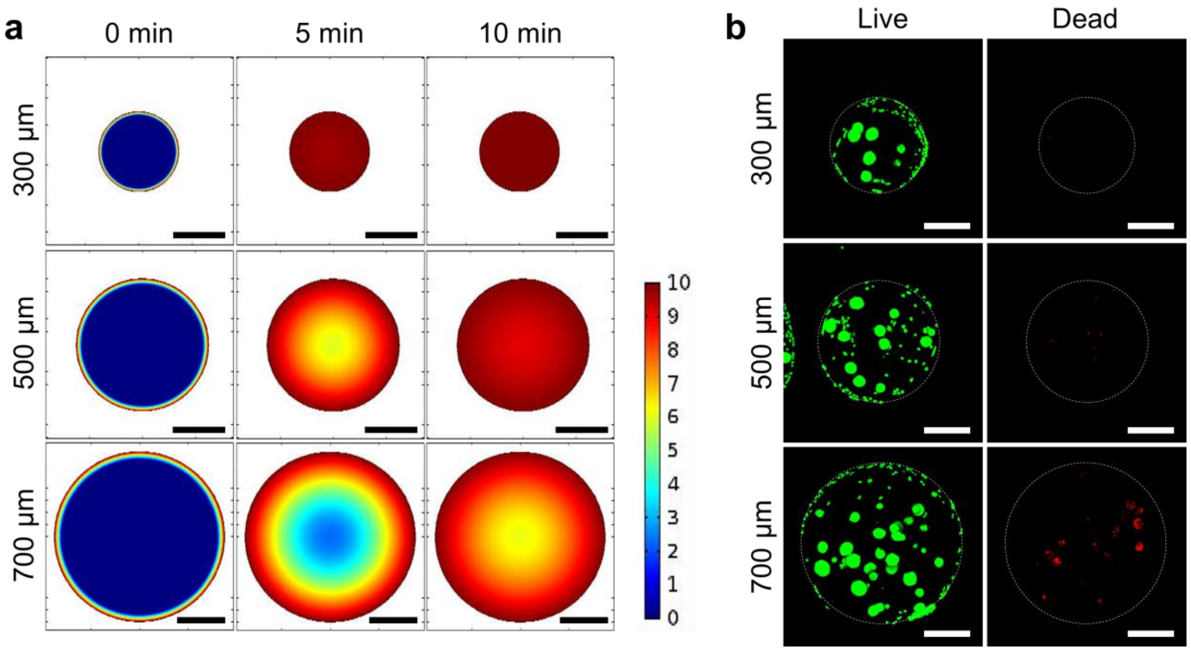

Supplementary Figure S3. Cell viability by the size of collagen beads. (a) Simulation of the diffusion of media, including nutrients, into differently sized collagen beads.[1] (b) Live/dead cell staining after 7 days of culture depending on the size of the collagen beads. Scale bars = 200 µm.

Recent study suggested the existence of spheroid heterogeneity in an *in vitro* model.[2] As the paper demonstrated, in the case of scaffold-free 3D culture (i.e., tumor spheroid), the use of the model as a drug screening platform has raised serious concerns about the reproducibility of the data produced, due to spheroid heterogeneity caused by the limited distribution of oxygen, nutrients, and metabolites, as well as a necrotic core. In fact, such phenomena could also be reproduced in a cell-laden matrix-based 3D tumor model. To avoid such heterogeneity, we systemically designed identical microbeads. To optimize the size and cell density per each microbead, we tested three different sizes of microbeads. After 7 days of culture, we analyzed Live/dead cell staining depending on the size of the collagen beads. In the microbead 700 µm in diameter, we observed decreased cell viability. The simulation pertaining to the diffusion of media in differently sized collagen beads suggested that the larger collagen beads were associated with inhomogeneous diffusion of media, including nutrients and metabolites. To avoid this limitation, we selected a diameter of 500 µm for our experiments.

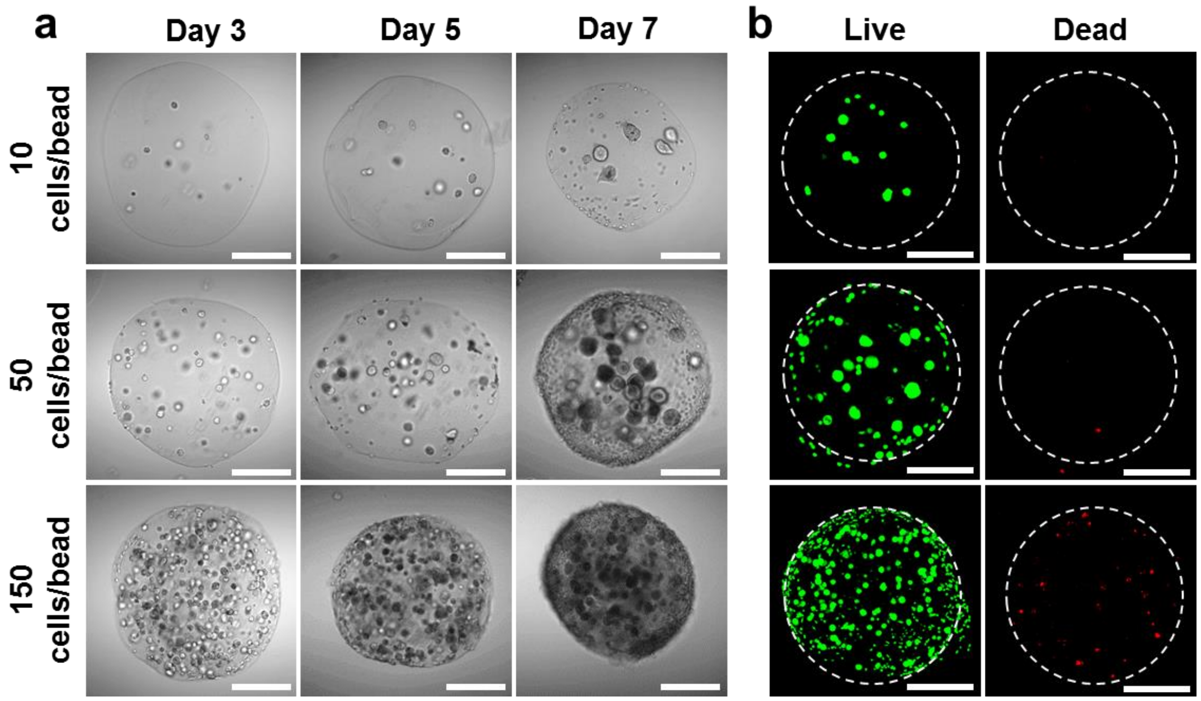

Supplementary Figure S4. Time-dependent cell culture and cell viability by cell concentration in 3D microbeads. (a) The density of the cells in the extracellular matrix (ECM) beads was controlled by modulating the initial cell concentration, which ranged from 10 to 150 cells per bead. (b) Cell viability depending on cell density after 5 days. Live (green) and dead (red). Scale bars = 200 µm.

To optimize cell density for each microbead, we employed three different densities (10 cells/bead, 50 cells/bead, and 150 cells/bead) with collagen beads of the same size (500 µm in diameter). As shown in Supplementary Figure S4, at relatively low cell densities (10 cells/bead and 50 cells/bead), AGS cells were efficiently encapsulated into the collagen beads. The encapsulated cells with a low density presented high viability. Upon increasing the cell density further to 150 cells/bead, the viability of encapsulated cells significantly decreased. This decreased viability presumably resulted from a deficiency of nutrients or oxygen in encapsulated cells in the center of the collagen beads during cell culture. A higher cell density may also enhance cell-to-cell contact, which could compensate for the reduced diffusion of nutrients.[3, 4] Therefore, we selected 50 cells/bead as a suitable concentration for further experiments.

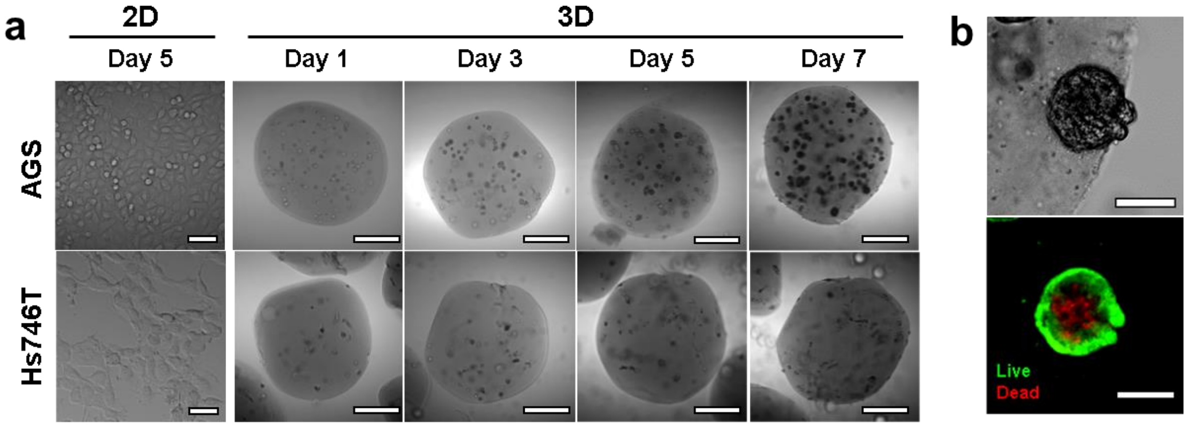

Supplementary Figure S5. (a) Time-dependent cell culture in 3D microbeads. In 3D culture, the gastric cancer cells were initially randomly distributed and evenly scattered in the collagen beads. During cell growth, the sparse cells became denser and more compact in the collagen beads. Scale bars = 200 µm. (b) Live/dead cell staining after 10 days. Cells were disseminated around the collagen beads and dead cells were observed in the center of cell aggregates after 10 days. Scale bars = 50 µm.

The duration of culture was set as 7 days because dead cells were observed in the center of the cell aggregation after 10 days (Supplementary Figure S5b).

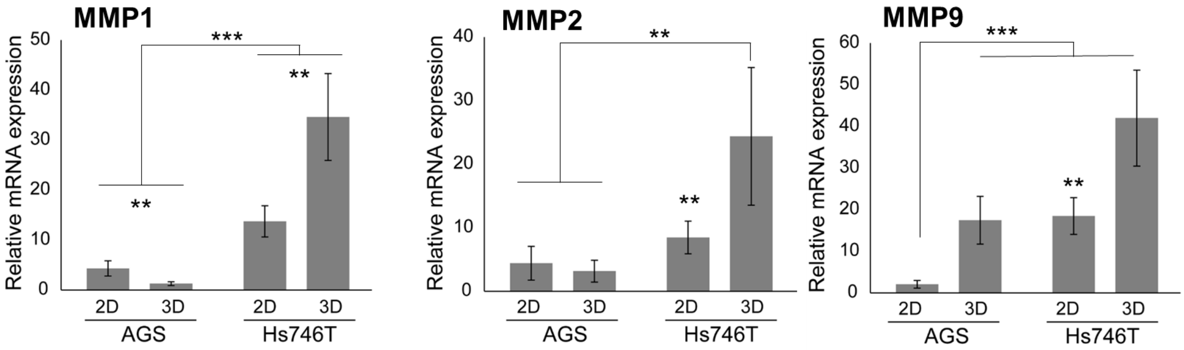

Supplementary Figure S6. Relative mRNA levels of matrix metalloproteinases (MMPs) in 2D and 3D platforms. MMP1 is related to the degradation of collagen type 1, and MMP2 and MMP9 are related to metastasis in gastric cancer. In AGS, MMP9 was increased in 3D microtumors; however, MMP1 and MMP2 did not show large differences. On the other hand, the expression levels of all MMPs in Hs746T increased in 3D microtumors. When comparing the two GC cell lines, diffuse Hs746T showed greater expression of MMPs than intestinal AGS. **: $p<0.01$, ***: $p<0.001$.

The signature of the diffuse type involved the altered expression of genes related to cell-matrix interaction, whereas that of the intestinal type involved the enhancement of cell growth. Thus, the secretion of factors involved in ECM remodeling, such as matrix metalloproteinases (MMPs), may be enhanced in Hs746T cells in collagen beads. In terms of the histological features, the Hs746T cells were characterized by single-cell migration. During the single-cell migration, the ability of the cells to deform relative to remodel tissues though proteolysis by MMPs.[5] This determines the efficiency of migration in 3D ECM. Most MMPs, including MMP-2 (gelatinase A, 72-kDa type IV collagenase), MMP-9 (gelatinase B, 92-kDa type IV collagenase), and MMP-1 (interstitial collagenase, collagenase-1), play significant roles in tumor invasion and metastasis. To clarify this point, we investigated the expression of these three MMPs in two different gastric cancer cell lines. From our analysis, the difference between 2D and 3D cultures was more significant for Hs746T cells than for AGS cells (Supplementary Figure S6). The expression of MMPs significantly increased in 3D-cultured Hs746T cells, while only MMP-9 was slightly increased in 3D-cultured AGS cells. This indicates that the 3D-cultured Hs746T cells actively remodel the ECM microenvironment to promote cell invasion and impact tumorigenesis and metastasis.

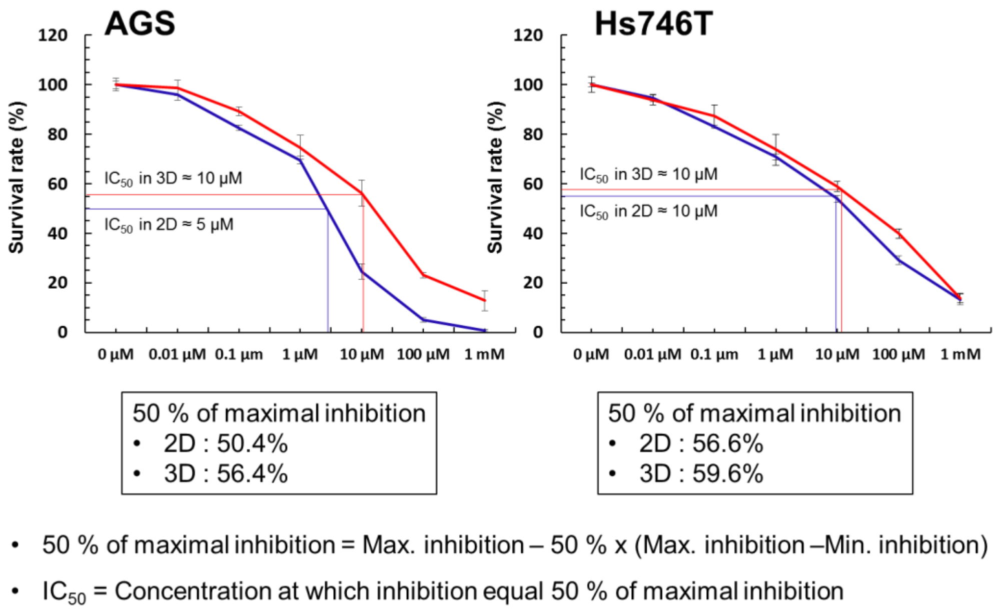

Supplementary Figure S7. Half-maximal inhibitory concentration (IC_{50}) in 2D and 3D cultures of AGS and Hs746T. IC_{50} means the concentration at which inhibition equals 50% of maximal inhibition. The value of 50% of maximal inhibition is calculated as follows: “Max. inhibition – 50% × (Max. inhibition – Min. inhibition)”. In our data, 50% of maximal inhibition in 2D- and 3D-cultured AGS was associated with 50.4% and 56.4% survival rates, respectively. In the case of Hs746T, 50% of maximal inhibition corresponded to 56.6% and 59.6% survival rates. According to the values of 50% of maximal inhibition in AGS, the values of IC_{50} are 5 and 10 μM in 2D and 3D cultures, respectively. In the case of Hs746T, the value of IC_{50} is approximately 10 μM in both conditions.

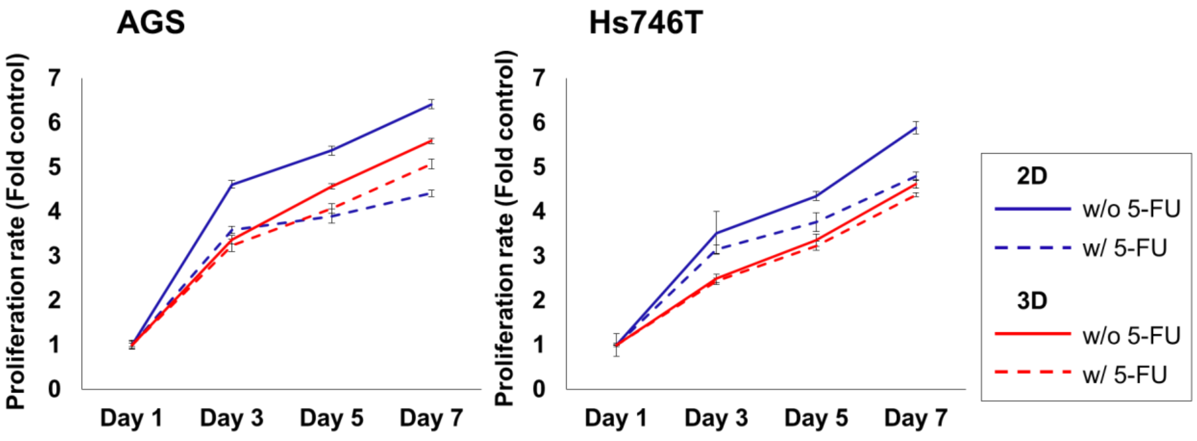

Supplementary Figure S8. Comparison of proliferation rates in 2D and 3D cultures upon drug treatment. We treated gastric cancer cells with 5-fluorouracil (5-FU) for 7 days based on the IC_{50} of the 2D-cultured AGS at 5 μM. AGS cells showed 68.8% and 90.6% survival upon drug treatment in 2D and 3D cultures. However, Hs746T showed no major difference in survival between 2D and 3D cultures, compared with AGS. Hs746T cells showed 87.2% and 94.7% survival after drug treatment, compared with the untreated control. A slight increase in survival of up to 7.5% was shown in the 3D microtumors compared with the 2D culture. The survival after drug treatment is calculated as follows: (proliferation rate under drug-treated conditions at 7 days / proliferation under untreated conditions at 7 days) × 100%.

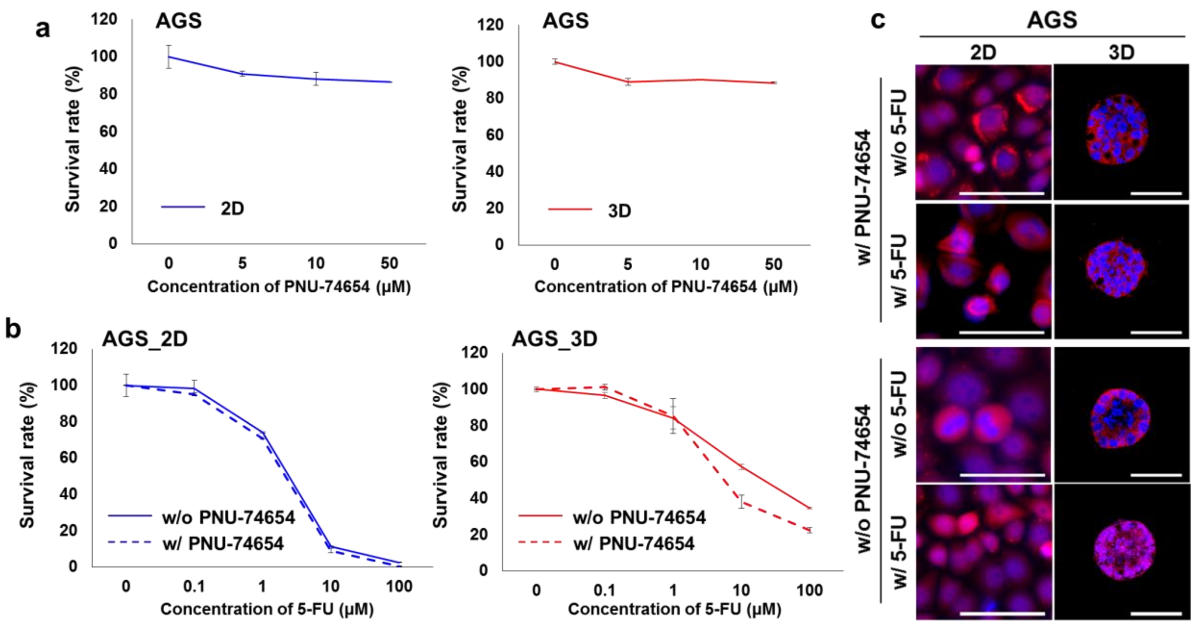

Supplementary Figure S9. Effects of the β-catenin inhibitor, PNU-74654, on 5-FU sensitivity in 2D and 3D AGS cultures. (a) Dose-dependent proliferation rates were assessed in the presence of PNU-74654. Cell proliferation was above 80% in 2D (blue) and 3D (red) cultures with 50 µM of PNU-74654. (b) Survival rates of 2D (blue) and 3D (red) AGS cultures treated with 5-FU following co-treatment with (dotted line) and without (straight line) 50 µM of PNU-74654. PNU-74654 affected drug resistance in 3D AGS cultures. (c) Immunofluorescence images of β-catenin with 5-FU and PNU-74654. β-catenin was translocated to the nucleus by 5-FU, which was inhibited by PNU-74654. Scale bars, 50 µm.

Detailed explanations on the relationship between EMT and N-cadherin expression in cancer cells

In general, EMT is a process in which epithelial cells lose their characteristic polarity, disassemble cell-cell junctions, and become more migratory.[6] Cadherin switching is a process in which cells shift to express different isoforms of the cadherin transmembrane proteins that underpin adherens junctions. Cadherins mediate cell-cell adhesion through their extracellular domains and connect to the actin cytoskeleton by associating with catenins through their cytosolic domains. Epithelial cells typically express E-cadherin, whereas mesenchymal cells express various cadherins, including N-cadherin. Numerous clinical studies have shown that E-cadherin is often lost in tumors *in situ*. Mesenchymal cells, which are more motile and less polarized than epithelial cells, typically express N-cadherin. However, some cancer cells derived from the epithelium inappropriately express N-cadherin, and the upregulation of N-cadherin expression has been shown to promote motility and invasion. This loss of E-cadherin expression and gain of N-cadherin expression is reminiscent of the cadherin switching that probably underpins many of the phenotypic changes. Therefore, the increased N-cadherin expression in microtumors could indicate that the ECM microenvironment influences the mode and dynamics of cancer cell migration, such as EMT.

Reference

1. E. E. Antoine, P. P. Vlachos and M. N. Rylander, *PLoS One*, 2015, **10**, e0122500.
2. M. Zanoni, F. Piccinini, C. Arienti, A. Zamagni, S. Santi, R. Polico, A. Bevilacqua and A. Tesei, *Sci Rep*, 2016, **6**, 19103.
3. F. Wu, X.-J. Ju, X.-H. He, M.-Y. Jiang, W. Wang, Z. Liu, R. Xie, B. He and L.-Y. Chu, *J. Mater. Chem. B*, 2016, **4**, 2455-2465.
4. T. Rossow, J. A. Heyman, A. J. Ehrlicher, A. Langhoff, D. A. Weitz, R. Haag and S. Seiffert, *J Am Chem Soc*, 2012, **134**, 4983-4989.
5. P. Friedl and K. Wolf, *Nat Rev Cancer*, 2003, **3**, 362-374.
6. M. J. Wheelock, Y. Shintani, M. Maeda, Y. Fukumoto and K. R. Johnson, *J Cell Sci*, 2008, **121**, 727-735.
